# Cell cycle control of kinetochore assembly

**DOI:** 10.1080/19491034.2022.2115246

**Published:** 2022-08-29

**Authors:** Qianhua Dong, Fei Li

**Affiliations:** Department of Biology, New York University, New York, NY, USA

**Keywords:** Kinetochore assembly, cell cycle, centromere, CENP-A, phosphorylation, CCAN, KMN, CENP-T, CENP-C

## Abstract

The kinetochore is a large proteinaceous structure assembled on the centromeres of chromosomes. The complex machinery links chromosomes to the mitotic spindle and is essential for accurate chromosome segregation during cell division. The kinetochore is composed of two submodules: the inner and outer kinetochore. The inner kinetochore is assembled on centromeric chromatin and persists with centromeres throughout the cell cycle. The outer kinetochore attaches microtubules to the inner kinetochore, and assembles only during mitosis. The review focuses on recent advances in our understanding of the mechanisms governing the proper assembly of the outer kinetochore during mitosis and highlights open questions for future investigation.

## Introduction

Genetic information must be precisely transmitted to the next generation during cell division. To achieve this end, each chromosome must be duplicated and equally segregated into daughter cells during each cell cycle. Accurate chromosome segregation during mitosis relies on a large protein complex, the kinetochore, a term coined by Lester Whyland Sharp in 1934 [1]. The disc-shaped structure, which contains more than 100 protein subunits in human cells, assembles on a specialized chromatin domain known as the centromere. The kinetochore links chromosomes to microtubule polymers and plays a key role in controlling chromosome movements. It also serves as a hub for the signaling molecules required to control accurate chromosome segregation [2,3]. Under the electron microscope, the kinetochore appears as a two-domain structure at metaphase, consisting of the inner kinetochore and the outer kinetochore. The inner kinetochore is built on the centromeric chromatin and serves as a structural platform for outer kinetochore assembly. The inner kinetochore constitutively associates with centromeres. The outer kinetochore interacts with microtubules and plays an essential role in generating and sensing microtubule attachments [2,4,5]. When cells enter mitosis, the outer kinetochore is quickly assembled on the platform of the inner kinetochore proteins. At the end of mitosis, the outer kinetochore is rapidly disassembled [6–8]. The kinetochore thus is a dynamic structure and tightly regulated over the course of the cell cycle. However, the molecular mechanisms underlying cell cycle-dependent kinetochore assembly are still not well understood. This review focuses on recent progress on our understanding of how the kinetochore assembly is cell cycle regulated. Due to space limitations, we limit our discussion to kinetochore assembly in mitosis. We refer readers interested in the reorganization of the kinetochore during error correction and meiosis to excellent reviews on these topics [3,9,10].

## Centromeres

Centromeres are a specific chromatin structure that is physically linked to the spindle via kinetochores during cell division. It was first defined by Walther Flemming in 1882 as the primary constrictions on chromosomes [11]. The identity of centromeres has not been revealed until recently. Most eukaryotes have large ‘regional centromeres’, which usually consist of AT-rich DNA repeats. The size and sequence of regional centromeres vary significantly across species, spanning from several kilobases to multiple megabases [12,13]. However, the underlying DNA sequence in centromeres is neither necessary nor sufficient for the centromere formation. Neocentromeres are new centromeres that are ectopically formed at non-centromeric regions when the native centromere is inactivated. Neocentromeres can arise both naturally and by experimental manipulation (for review, see [14,15]), providing strong evidence that centromere formation is an epigenetic event. Recent studies revealed that the regional centromere is epigenetically defined by a conserved centromeric specific histone H3 variant, CENP-A. CENP-A replaces its canonical counterpart and forms specific CENP-A nucleosomes with histone H4, H2A, and H2B, which are interspersed with canonical histone H3-containing nucleosomes in centromeres [16–21]. Mislocalized CENP-A recruits kinetochore proteins to non-centromeric regions, leading to chromosome missegregation defects in a variety of organisms (for review, see [22]). The histone fold domain of CENP-A contains the conserved CENP-A targeting domain (CATD), which is necessary and sufficient for centromeric localization of CENP-A in vertebrates [23,24].

Loading of CENP-A to centromeres is mediated by the conserved histone chaperone HJURP/Scm3 in a cell cycle-dependent manner [25–32]. In metazoans, loading of new CENP-A is restricted in the G1 phase of the cell cycle. The recruitment of HJURP to centromeres is mediated by the Mis18 complex, which is composed of Mis18α, Mis18β, and Mis18BP1. The cyclin-dependent kinases 1 and 2 (Cdk1/2) phosphorylate HJURP and Mis18BP1 during S and G2-phase, inhibiting premature CENP-A loading during this time [33–35]. On the other hand, the Polo-like kinase 1 (PLK1) phosphorylates the Mis18 complex during G1 to promote the deposition of new CENP-A to centromeres [36].

Transcription of CENP-A is also cell cycle regulated to ensure that optimal level of CENP-A is generated (for review, see [22]). In fission yeast, the temporal control of CENP-A^Cnp1^ transcription is mediated by the MBF (MluI box-binding factors) complex, which consists of Res1, Res2, Cdc10, Nrm1, and Yox1. The periodic transcription of CENP-A^Cnp1^ is lost in MBF mutants, resulting in the higher level of CENP-A^Cnp1^ and chromosome segregation defects [37]. A recent study has also shown that the Cdk5 regulatory subunit-associated protein 2 (Cdk5rap2) acts as a positive transcriptional regulator of CENP-A in human cells [38]. Regional centromeres are typically buried in a large pericentric heterochromatin, a condensed and transcriptionally inert chromatin domain lacking CENP-A [39,40]. Heterochromatin, which is epigenetically defined by histone H3 lysine 9 (H3K9) methylation, contributes to the formation of regional centromeres [41–48].

On the other hand, the ‘point’ centromere in the budding yeast *Saccharomyces cerevisiae* is genetically determined by a 125-bp DNA sequence, which contains three centromere-determining elements (CDEI, CDEII, and CDEIII) [49–51]. The budding yeast centromeres also contain a CENP-A homolog, Cse4. CENP-A^Cse4^ is important for kinetochore assembly and chromosome segregation [52–54] and can functionally replace the human CENP-A [55]. The deposition of CENP-A^Cse4^ to centromeres is facilitated by the centromere DNA-binding complex, CBF3 [54,56,57]. In addition, some species in worms, plants, and insects use the whole chromosome as the centromere, which is called the ‘holocentric chromosome’ [58].

## The inner kinetochore

**Figure 1.**
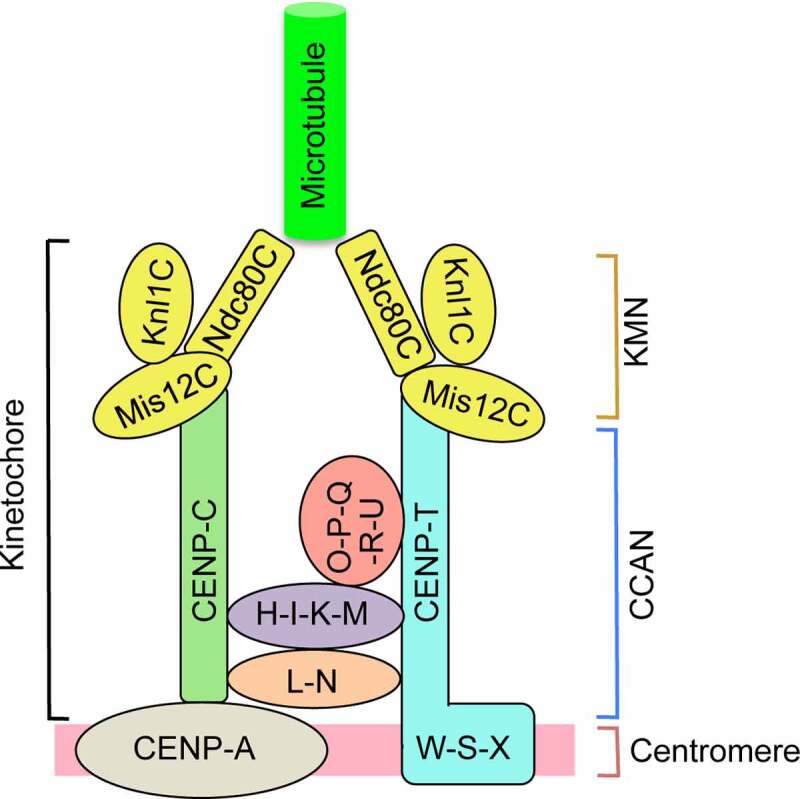
**A schematic of a canonical kinetochore**. The main structure of the kinetochore consists of constitutive centromere-associated network (CCAN), which includes five subgroups (CENP-L-N, CENP-H-I-K-M, CENP-O-P-Q-R-U, CENP-T-W-S-X and CENP-C), and the KMN (Knl1, Mis12, and Ndc80 complexes) network. Kinetochore position is specified by CENP-A-containing nucleosomes, upon which CCAN assembles. CCAN recruits KMN, which directly binds microtubules, during mitosis. Two independent pathways, CENP-C and CENP-T, link KMN to CCAN.

The inner kinetochore consists of conserved 16 proteins in vertebrates, collectively known as constitutive centromere-associated network (CCAN). CCAN was originally identified by affinity purification of CENP-A-containing nucleosomes [59]. CCAN constitutively binds to the centromere throughout the cell cycle and can be divided into five subgroups, including CENP-L-N, CENP-H-I-K-M, CENP-O-P-Q-R-U, CENP-T-W-S-X, and CENP-C (Figure 1). CENP-C is a long disordered protein and forms a dimer through the C-terminal Cupin domain [60,61]. It interacts directly with the CENP-A nucleosome through the central domain and the short CENP-C motif [62–65]. CENP-C has multiple contacts with the structure of CCAN, including CENP-L-N and CENP-H-I-K-M, and is required for centromeric localization of other CCAN components [60,65–68], and thus serves as a hub for the assembly of the inner kinetochore. The CENP-L-N complexes also have been shown to directly interact with CENP-A nucleosomes [69,70]. However, a recent structural study using the full human CCAN complex assembled on the CENP-A nucleosome revealed that CENP-L-N interacts with the linker DNA but not the CENP-A nucleosome [68]. It is possible that CENP-L-N has different binding modes depending on cellular states. The CENP-L-N subgroup serves as a structural keystone of CCAN. CENP-H-I-K-M and CENP-O-P-Q-R-U are assembled on either side of the CENP-L-N [68,70].

The CENP-T-W-S-X consists of two heterodimers, CENP-T-W and CENP-S-X. Each subunit in the CENP-T-W-S-X contains the histone fold domain [71,72]. The CENP-T-W in fission yeast and vertebrates is essential for viability, but CENP-S-X is not [66,73–75]. Structural studies revealed that human CENP-T-W-S-X has multiple interactions with neighboring CCAN subunits, including CENP-H-I-K-M, CENP-L-N, and CENP-O-P-Q-R-U [68]. In addition to the histone fold domain, CENP-T also contains a conserved histone fold extension (HFE) motif at the C-terminus and a long unstructured N-terminus. The histone fold domain of CENP-T forms a dimer with CENP-W, whereas the HFE motif interacts with the CENP-H-K-I-M [71,76]. The CENP-T-W-S-X also directly binds centromeric DNA. The DNA binding activity of the complex depends on the histone fold domain of CENP-T and CENP-W and is essential for kinetochore formation [71,74]. Chicken CENP-T-W-S-X is assembled into a stable heterotetramer in vitro and has been proposed to form a nucleosome-like structure [71]. This was supported by a recent structural study using the full human CCAN with α–satellite DNA [68]. The CENP-T-W-S-X also appears to bind histone H3, not to CENP-A [74,77]. It is believed that deposition of CENP-T is independent of CENP-A, but the association of CENP-T with centromeres is strongly reduced in CENP-A knockout cells [74,78–80]. Whole-proteome genetic analysis of chicken DT40 cells suggested that CENP-T depends on CENP-N for its centromeric localization [81]. A recent study also showed that RbAp48/RbAp46^Mis16^ is required for the deposition of CENP-T to centromeres in fission yeast [82]. Unlike CENP-T-W in vertebrates, CENP-T^Cnn1^ in budding yeast is exactly colocalized with CENP-A^Cse4^ nucleosomes [83,84], suggesting that CENP-T-W-S-X in budding yeast is unlikely to form nucleosome-like structure. The centromeric localization of CENP-T^Cnn1^ depends on CENP-I^Ctf3^ [84].

Although CCAN components localize constitutively at centromeres, the level of individual components, the timing of new protein loading at centromeres, and physical and functional relationships between different sub-groups can change throughout the cell cycle. Thus, the association of CCAN components with centromeres can still quite dynamic (for review, see [8]). The mechanisms underlying the dynamic behavior of CCAN during the cell cycle remain poorly understood.

## The outer kinetochore

The outer kinetochore is assembled on the platform of inner kinetochore proteins during mitosis and directly binds to microtubules. The 10-subunit outer kinetochores are categorized into three complexes: the Ndc80 complex (Ndc80C), the Mis12 complex (Mis12C), and the Knl1 complex (Knl1C). The three complexes form the KMN (Knl1C, Mis12C, and Ndc80C) network [2,85,86] (Figure 1). The Ndc80C contains four subunits: Ndc80, Nuf2, Spc24, and Spc25, and serves as the primary microtubule receptor on the kinetochores. The four subunits form two heterodimers: Ndc80-Nuf2, and Spc24-Spc25 [87–89]. The Ndc80-Nuf2 heterodimer contains a globular N-terminal region and a C-terminal coiled coil, while the Spc24-Spc25 heterodimer contains a globular C-terminal region and an N-terminal coiled coil. Each pair of proteins interacts via the coiled coil regions to form a tetramer, giving an overall dumbbell-like shape with a globular domain at each end. The Ndc80C binds microtubules via the globular N-terminal region of Ndc80-Nuf2, known as the MT-binding module containing calponin-homology (CH) domains [90–92]. The globular C-terminal region of Spc24-Spc25 contains RWD (RING finger and WD-repeats) domains, which interact with the disordered N-terminus of CENP-T [93–96]. The Mis12C consists of Mis12, Nnf1, Nsl1, and Dsn1. The complex is roughly rod-shaped, and binds both Ndc80C and Knl1C [95,97,98]. The Mis12C also interacts with CENP-C and CENP-T and serves as an interaction hub between the inner kinetochore and the outer kinetochore [97,99,100]. The Knl1C is composed of two subunits, Knl1 and ZWINT. Knl1, the largest subunit in the outer kinetochore, contains RWD domains at its C-terminus that directly binds the Mis12C [98,101]. ZWINT is involved in the spindle assembly checkpoint (SAC), a surveillance mechanism that prevents defects in chromosome segregation [102,103]. Interestingly, the KMN network components are conserved and exist in most eukaryotes, whereas the CCAN components are highly divergent across species [104].

## Pathways linking the inner kinetochore to the outer kinetochore

Assembly of the outer kinetochore on the inner kinetochore during cell division is a crucial step in establishing microtubule attachments to the kinetochore. CENP-C and CENP-T are the two major pathways that connect the inner kinetochore with the outer kinetochore [5,7] (Figure 1). Artificial tethering of partial CENP-C and CENP-T into a noncentromeric region indicated that CENP-C and CENP-T form two parallel pathways for the recruitment of the KMN network onto the kinetochore [100,105].

CENP-C exists largely as an elongated protein that may span >100 nm at the kinetochore [106]. The multi-domain protein not only serves as a hub for inner kinetochore assembly but also directly binds the Mis12C via its N-terminal region. CENP-C can recruit one copy of the Ndc80C through its interaction with Mis12 [65,99,100,107,108]. Artificial targeting of the N-terminus of CENP-C to a non-centromeric locus in human and chicken cells results in recruitment of KMN [100,105,107].

CENP-T binds Ndc80C through its extended unstructured N-terminal region. The Ndc80-binding domain at CENP-T forms an alpha-helix that directly interacts with the Spc24-Spc25 heterodimer in Ndc80C [93,94]. The CENP-T N-terminal region also directly interacts with Mis12C, which in turn binds Ndc80C [96]. Artificial tethering of the N-terminus of CENP-T in vertebrate cells is sufficient to recruit KMN to a non-centromeric locus [105]. Human CENP-T has two Ndc80C binding sites, whereas chicken CENP-T only has one site. In addition, CENP-T can indirectly recruit one copy of Ndc80C through its interaction with Mis12C [109,110]. Thus, a total of two copies of the Ndc80C exist on each CENP-T in chicken cells via either direct binding or Mis12C, but three copies for human CENP-T. A recent study in chicken cells demonstrated that both copies of the Ndc80C on chicken CENP-T are required for establishing proper kinetochore–microtubule interaction [109]. Configuration of two copies of Ndc80C has also been suggested for budding yeast CENP-T^Cnn1^ [84]. Artificial tethering assays using various CENP-T mutants in human and chicken cells demonstrated that the direct binding of Ndc80C to the CENP-T N-terminus is required for the CENP-T-Mis12C interaction, suggesting that the direct CENP-T-Ndc80C interaction acts upstream of Mis12C-recruitment to CENP-T [105,107].

The choice of different pathways linking the inner kinetochore and the outer kinetochore is different among different species. In human cancer cell lines, both CENP-C and CENP-T genes are essential for cell growth and proliferation [111], suggesting that both pathways are vital for kinetochore assembly. In chicken cells, although the N-terminus of CENP-C is essential for its interaction with Mis12C, CENP-C-Mis12C interaction is dispensable for cell viability while CENP-T is essential [109,110]. In fission yeast, deletion of CENP-C^Cnp3^ does not result in cell death, but CENP-T^Cnp20^ is indispensable [66], suggesting that the CENP-T pathway plays a more dominant role in kinetochore assembly in these organisms. On the other hand, although CENP-T^Cnn1^ and CENP-C^Mif2^ are conserved in budding yeast, CENP-T^Cnn1^ is not essential for cell viability, whereas CENP-C^Mif2^ is indispensable for chromosome segregation [112–114]. Further analysis revealed that, though CENP-A recognition by CENP-C^Mif2^ is essential for its kinetochore function, its binding to Mis12^Mtw1^ is dispensable [115]. In addition, a third pathway mediated by CENP-U^Ame1^ in budding yeast is used for recruiting KMN to CCAN. CENP-U^Ame1^ has the Mis12^Mtw1^ complex (Mis12^Mtw1^C)-binding domain containing the first 15 amino acids (aa) in its N-terminus, and deletion of the domain causes cell lethality, suggesting that the CENP-U^Ame1^ pathway is the main pathway for kinetochore assembly in budding yeast [115,116]. CENP-U^Ame1^ also has a homolog in other species, including CENP-U in human and chicken and CENP-U^Mis17^ in fission yeast [94]. Whether CENP-U in these species is involved in KMN recruitment needs further characterization. Interestingly, in some species, such as *Drosophila melanogaster* and *Caenorhabditis elegans*, the CENP-T pathway is lost during evolution, and the CENP-C pathway alone is responsible for the assembly of KMN on the inner kinetochore [108,117–121]. On the other hand, holocentric Lepidoptera (butterflies and moths) lacks both CENP-A and CENP-C homologs [122]. A recent study showed that CENP-T in these insects is sufficient to recruit Mis12C and Ndc80C [123]. The diversity of inner kinetochore architecture may be explained by the coevolution of inner kinetochore proteins and rapidly changing centromeric DNA sequences [104].

## Cell cycle regulation of kinetochore assembly

The inner kinetochore proteins are constitutively localized at centromere in all the stages of the cell cycle, while the outer kinetochore are only assembled at centromeres during mitosis in vertebrates. Nevertheless, in both fission yeast and budding yeast, outer kinetochore proteins, such as Ndc80, appear to associate with centromeres through the different stages of the cell cycle [87,124]. However, a recent work by Jiménez-Martín et al. demonstrated that the outer kinetochore is indeed also reassembled at the onset of mitosis in fission yeast, similar to metazoans [125]. Fission yeast chromatin is characterized by the evolutionarily conserved Rabl chromosome configuration during interphase, in which centromeres are clustered underneath the nuclear envelope near the spindle pole body (SPB) and telomeres are also attached to the nuclear envelope [126]. The recent study showed that when Rabl configuration is removed, the outer kinetochore reassembly was then observed during mitosis, indicating that Rabl configuration masks kinetochore reassembly in fission yeast [125]. Consistent with this, Dong et al. showed that Ndc80 and CENP-T^Cnp20^ strongly interact during mitosis but not interphase [82]. Similarly, in budding yeast, the interaction between Ndc80C and CENP-T^Cnn1^ also occurs predominantly in mitosis, suggesting that the outer kinetochore adopts a structural change during the stage of the cell cycle [94]. These works indicated that outer kinetochore disassembly/assembly program during cell cycle progression is a conserved phenomenon. Recent studies have demonstrated that post-translational modifications, such as phosphorylation, play a crucial role in governing the interaction between CCAN and KMN during mitosis.

### Phospho-regulation of outer kinetochore assembly through the cell cycle

CENP-T phosphorylation has been well studied and found to be conserved among different species. The N-terminus of CENP-T contains the Ndc80C-binding domain, which binds the Spc24-Spc25 heterodimer in Ndc80C. Phosphorylation of the Ndc80C-binding domain in CENP-T by Cdk1 stabilizes the interaction [93,94]. In human cells, Cdk1-mediated phosphorylation at Thr11 and Thr85, both of which reside in the conserved Cdk1 consensus sequence within the Ndc80C-binding domain, promotes the recruitment of two copies of Ndc80C to CENP-T [96,107]. Chicken CENP-T has one Cdk1 consensus sequence in the Ndc80C-binding domain, and phosphorylation of Thr72 within the sequence is important for recruitment of Ndc80C to CENP-T [93]. Budding yeast CENP-T^Cnn1^ also contains a conserved Ndc80C binding motif at its N-terminus [94]. The N-terminus of CENP-T^Cnn1^ can be phosphorylated by both Cdk1 and mitotic kinase Mps1. However, Cdk1 is not required for the interaction of CENP-T^Cnn1^ and Ndc80C. Instead, Mps1-mediated Ser74 phosphorylation contributes to the interaction [95,127]. The interaction between Ndc80C and CENP-T^Cnn1^ in budding yeast is not essential, while the Ndc80C-Mis12^Mtw1^C interaction is. It has been proposed that Mps1 promotes the formation of essential Ndc80C-Mis12^Mtw1^C during S phase and early mitosis by inhibiting the Ndc80C and CENP-T^Cnn1^ interaction [95].

Cdk1-mediated phosphorylation also plays an important role in CENP-T-Mis12C interaction. The aa 201–230 region in human CENP-T is critical for Mis12C recruitment. Thr195 and Ser201 in this region have been shown to be critical for recruitment of Mis12C to CENP-T [96,107]. However, in chicken cells, a phospho-null mutant of Thr184 (corresponding to human CENP-T S201) is viable [110]. In fact, the phospho-null mutant of all the Ser/Thr from aa 161 to 216 in the Mis12C binding region does not have any defect [109]. These data suggest that an additional regulatory mechanism controls the CENP-T-Mis12C interaction in chicken cells.

In addition, the CENP-C-Mis12C interaction is also mediated by mitotic phosphorylation. Dsn1 in the Mis12C can auto-inhibit the CENP-C-Mis12C interaction via its conserved basic domain by masking the CENP-C-binding interface in Mis12C [128,129]. Aurora B kinase, which is known to be involved in error correction and SAC response, phosphorylates the basic domain of Dsn1 during mitosis. The phosphorylation results in an increased binding affinity between CENP-C and Mis12C in vitro and centromeric localization of Mis12C in mitotic cells [128,130,131].

Hara et al. also showed that Cdk1-mediated phosphorylation contributes to the Mis12C-Ndc80C interaction [110]. Dsn1 in Mis12C binds Ndc80C through its C-terminus, which contains a sequence similar to the Ndc80C-binding region in the CENP-T N-terminus. The Ndc80C-binding region in Dsn1 contains one Cdk1 consensus sequence [110,128]. Interestingly, while phosphorylation of CENP-T by Cdk1 stabilizes its interaction with Ndc80C, Cdk1-mediated phosphorylation in the Ndc80-binding region of Dsn1 decreases its binding affinity with Spc24-Spc25, leading to unstable interaction between Mis12C and Ndc80C. Thus, Cdk1 phosphorylation is a negative regulator of Mis12C-Ndc80C interaction, which may explain why a majority of Ndc80C localized to the CENP-T pathway, but less to Mis12C on the CENP-C pathway during mitosis in chicken DT40 cells [110].

The kinetochore-microtubule attachment is stabilized by the Ska complex, which contains three subunits, Ska1, Ska2, and Ska3 [132–134]. During mitosis, Ska3 is also phosphorylated by Cdk1. Phosphorylated Ska3 binds to Ndc80C which in turn recruits the Ska complex to kinetochores. Ska3 mutants lacking Cdk1 phosphorylation are defective in kinetochore localization [135,136].

### Mutually exclusive binding modes establish distinct configurations of the outer kinetochore

A recent study in fission yeast revealed that phosphorylation-mediated competitive exclusion of different proteins at the N-terminus of CENP-T also regulates the recruitment of KMN. Fission yeast contains regional centromeres, epigenetically defined by CENP-A^Cnp1^ [137,138]. Dong et al. previously identified that Ccp1, a conserved nucleosome assembly protein (NAP) domain-containing protein, antagonizes the loading of CENP-A in both centromere and non-centromeric regions in fission yeast [139]. Interestingly, Ccp1 associates with centromeres during interphase but dissociates from centromeres during mitosis [139,140]. The biological significance of this cell cycle-regulated association of Ccp1 with centromeres is unknown. Dong et al. recently revealed that Ccp1 binds to CENP-T^Cnp20^ and further showed that CENP-T^Cnp20^ is required for Ccp1 centromeric localization [82].

**Figure 2.**
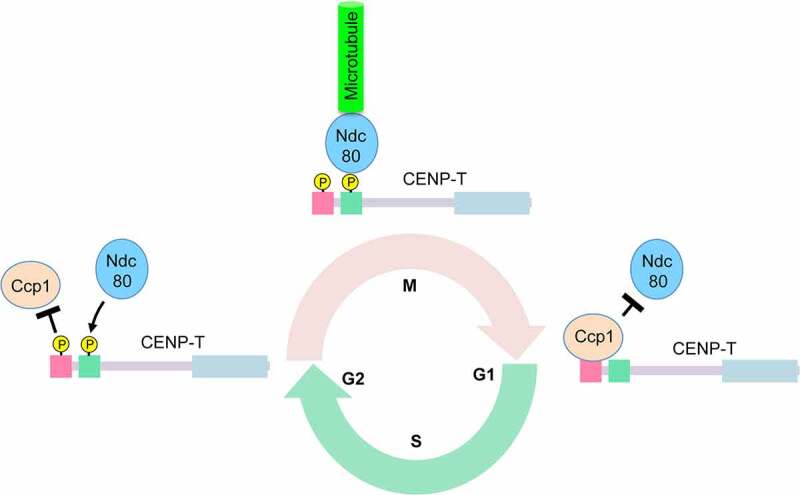
**Phosphorylation-mediated competitive exclusion between Ccp1 and Ndc80 at the N-terminus of CENP-T regulates the recruitment of KMN**. The Ccp1-binding domain of CENP-T is localized adjacent to the Ndc80-binding domain at the N-terminal region of CENP-T. When cells enter mitosis, the Ccp1-binding domain of CENP-T is phosphorylated by the Cdk1 kinase. Phosphorylation of the Ccp1-binding domain dissociates Ccp1 from CENP-T, allowing Ndc80C to bind to the Ndc80-binding domain. Ndc80C then directly interacts with microtubules to facilitate chromosome segregation. When cells exit from mitosis, the Ccp1-binding domain is dephosphorylated, which recruits Ccp1. Reassociation of Ccp1 with the Ccp1-binding domain blocks the binding of Ndc80C to CENP-T during interphase. P: phosphorylation.

CENP-T^Cnp20^ in fission yeast is essential for viability and contains a Ndc80-binding region (aa 70–87) at its N-terminus. The Ndc80 binding region contains a conserved Cdk1 consensus sequence [94]. Ccp1 binds to the first 1–55 amino acids of CENP-T^Cnp20^, which is immediately adjacent to the Ndc80-binding region. The Ccp1-binding motif in CENP-T^Cnp20^ contains four conserved Cdk1 consensus sequences and can be phosphorylated by Cdk1 in vitro [82]. Phosphorylation of the Ccp1-binding motif in CENP-T^Cnp20^ decreases its interaction with Ccp1. Consistent with this, in the phosphomimetic mutant of the Ccp1 binding motif, Ccp1 dissociates from centromeres through all stages of the cell cycle. In contrast, Ccp1 associates with centromeres during both mitosis and interphase in the phospho-null mutant of the motif. Furthermore, the phospho-null mutant of Ccp1-binding motif disrupts the positioning of Ndc80C during mitosis and displays severe chromosome missegregation defects, suggesting that occupancy of Ccp1 at the Ccp1-binding domain in CENP-T prevents the interaction of the adjacent Ndc80-binding domain with Ndc80C [82]. Together, this study suggests the following model: at the onset of mitosis, Cdk1 mediated phosphorylation of CENP-T^Cnp20^ at the Ccp1-binding motif ejected the Ccp1 and makes the room for the binding of Ndc80C to CENP-T^Cnp20^; at the end of mitosis, the Ccp1-binding motif is dephosphorylated, leading to the recruitment of Ccp1 that blocks the binding of Ndc80C to CENP-T (Figure 2). Phosphorylation-mediated competitive exclusion between Ccp1 and Ndc80C represents a new mechanism governing the cell cycle-dependent kinetochore assembly. The principle explains the observation that Ccp1 dissociates from centromeres during mitosis.

## Concluding remarks

The kinetochore plays a fundamental role in accurate chromosome segregation during mitosis. The large protein machine must be precisely assembled on centromeric chromatin during mitosis to generate a microtubule-binding interface that links chromosomes to the mitotic spindle. Kinetochore dysfunctions can lead to chromosomal instability (CIN) and aneuploidy, both of which are common characteristics of cancer cells [141–143]. However, in spite of recent achievements in understanding the components and functions of the kinetochore, there remains much to be learned about how kinetochore assembly is cell cycle regulated. Phosphorylation has been heavily studied as regulators of the events leading to kinetochore assembly. The role of other posttranslational modifications in kinetochore assembly during mitosis is less well understood. Future studies are needed to address this issue. A variety of different strategies were adopted by different species to recruit KMN to the inner kinetochores. A detailed understanding of how CENP-C and CENP-T pathways are used and coordinated to recruit KMN in these organisms will shed important light on the kinetochore’s role in chromosome segregation. Phosphorylation-mediated competitive exclusion between Ccp1-Ndc80 provides a new insight into the cell cycle-dependent kinetochore assembly. Similar to Ccp1, multiple other centromeric proteins in fission yeast, including HJURP/Scm3, Mis16, Mis18, Eic1/Mis19/Kis1, and Eic2/Mis20, also display the same distribution pattern through the cell cycle [26,28,29,144–147]. In addition, Mis18α in chicken DT40 cells is localized at centromeres during interphase, but is lost during mitosis [148]. It will be interesting to know whether these centromeric proteins use a similar mechanism to regulate kinetochore assembly. While much of the focus has been on understanding the assembly of kinetochores at the onset of mitosis, how kinetochores are disassembled at the end of mitosis have received less attention. The PP2A (protein phosphatase 2A), which is able to dephosphorylate CDK1 substrates during anaphase, has been implicated in kinetochore disassembly [6]. The molecular basis of PP2A-mediated kinetochore disassembly will need to be addressed in the future.
